# Evaluation of the toxic effect of star fruit on serum biochemical parameters in rats

**DOI:** 10.4103/0973-1296.62899

**Published:** 2010-05-05

**Authors:** Z. Y. Khoo, C. C. Teh, N. K. Rao, J. H. Chin

**Affiliations:** *Faculty of Pharmaceutical Sciences, UCSI University, Cheras, 56000, Kuala Lumpur, Malaysia*

**Keywords:** LD_50_, liver, oral toxicity, serum enzyme, star fruit

## Abstract

The objective of the present study was to evaluate the toxic effect of ***Averrhoa carambola*** (star fruit) juice at different storage conditions in Sprague Dawley (SD) rats. Twenty female rats weighing 180 ± 20 g were randomly assigned into four groups with five rats per group (*n* = 5). First group served as the control group, fed with distilled water (vehicle). Second, third and fourth groups were orally treated with juice of ***A. carambola*** stored for 0, 1 and 3 h respectively for 14 days. Cage-side observations were done daily after each treatment. Body weight, food consumption and water intake were recorded on day-0, day-3, day-7 and day-14. All rats were fasted overnight prior to blood collection through cardiac puncture on day-15. The levels of alanine aminotransferase (ALT), aspartate aminotransferase (AST), alkaline phosphatase (ALP), urea and creatinine in blood serum were measured. Data were analyzed using Dunnett's test. From the results obtained, there was no lethality found and LD_50_ could not be determined. Increment of ALT levels (*P*<0.05) was reported in those rats treated with ***A. carambola*** juice stored for 3 h. On the basis of these results, we can conclude that ***A. carambola*** juice stored for 0 hand 1 h are safe to be consumed. However, juice stored for 3 h exerts toxic effect on rat liver at hepatocellular level.

## INTRODUCTION

*Averrhoa carambola*, or locally known as star fruit, is a product of the carambola tree (Family: Oxalidaceae) which is short trunked with lots of branches, bushy, rounded crown with height reaches 6–9 m. Star fruit is becoming a popular fruit in tropical countries, such as Taiwan, southern China, India, Central America and Brazil.[1] Its transverse shape is similar to a star with five lobes of fruit flesh and is yellowish-green in color.[2] Sweet star fruits are also processed into wine. Star fruits are believed to be beneficial for its antioxidant property and rich nutrient content. Epicatechin and pro-anthocyanidins are the major phenolic compounds that are responsible for the antioxidant property of star fruit.[3–4]

Recently, a few cases have been reported on kidney failure patients experiencing hiccups, nausea, weakness, low back pain, confusions, fever and other symptoms or biochemical alteration after ingesting star fruit.[5] Since then, the public has started to question the safety of star fruit ingestion. Many researchers are trying to figure out the toxicity and identify the related substances in star fruit. The mechanism of the toxic effects of star fruit on humans remains unclear although oxalate and methanol are the most suspected substances that may cause severe toxicity in human as they are present in large amounts in star fruit and are toxic in nature.[6–7] Limited data for star fruit juice are available for the establishment of a LD_50_ and no-observed-adverse effect level (NOAEL) in laboratory study. The present oral toxicity study is conducted according to the Organization for Economic Cooperation and Development (OECD) Toxicity guideline. The main objectives of the present study are to evaluate the possible toxic effects of different storage conditions of *A. carambola* juice on liver and kidney functions in young SD male rat and to determine the LD_50_ of star fruit juice in animals.

## MATERIALS AND METHODS

### Star fruit juice preparation

Semi ripe star fruits purchased from a local hypermarket were used in the present experiment. All star fruits were kept in the refrigerator. About 100 g of the star fruit was used to extract the juice using a juice blender. The star fruit juice was filtered and the volume of juice extracted from 100 g of star fruit was recorded. The star fruit juice was kept at room temperature (25°C) until use. The star fruit juice at three different conditions were prepared: (i) freshly extracted star fruit juice, (ii) star fruit juice kept for 1 h after extraction and (iii) star fruit juice kept for 3 h after the extraction.

### Experimental animal selection

Twenty healthy young female Sprague-Dawley (SD) rats (14 ± 1 week old) (180 ± 20 g) bred in the Animal Transit Room Unit of UCSI University were chosen for the toxicity study. The animals were kept in the animal transit room with maintained temperature at 25 ± 2°C under 12 h–light/dark cycles. All experimental rats were freely accessible to tap water and food pallets during the acclimatization period. Animals were handled based on the guidelines of the animal ethics, Faculty of Pharmaceutical Sciences, UCSI University.

### Acute oral toxicity

All animals were randomly assigned into four groups with five animals per group (*n* = 5). First group served as the control group (treated with vehicle only i.e. distilled water). Groups 2–4 were treated once daily for 14 days with freshly prepared star fruit juice, and star fruit juice kept for 1 h and 3 h under room temperature after the extraction, respectively. Body weight of each rat was recorded each time before feeding. Water intake, food consumption and body weight change for the control and treatment groups were recorded at day-0, day-7 and day-14. Cage-side observation was conducted extensively every half an hour in the first 4 h and followed by every 6 h in the first 24 h. Any lethality to the rats, toxic symptoms and any adverse effects within 24 h post administration of star fruit extract were recorded. All rats were fasted overnight (at least 16 h prior to blood collection) after the last dose treatment at day-14. At day-15, all rats were anesthetized by diethyl ether inhalation and blood was taken *via* cardiac puncture by using a needle (size 0.50 × 16 mm, Terumo) to collect about 1.0-1.5 ml of blood.[8] Blood serum samples were sent to Elite Pathology Laboratory for analysis within the day of experiment. Several serum biochemical parameters, that is alanine aminotransferase (ALT), aspartate aminotransferase (AST), alkaline phosphatase (ALP), urea (BUN), creatinine, were determined.

### Data analysis

All data were expressed as mean ± standard deviation and analyzed using Dunnett's test. *P*<0.05 and *P*<0.01 were considered to be significant when compared to the respective control group.

## RESULTS AND DISCUSSION

Oral administration of *A. carambola* juice did not cause any abnormal changes and observable toxic symptoms such as tremor, righting reflex, ataxia as well as other nervous responses. No significant change in terms of change in mean body weight, food and water intake was observed between rats treated with star fruit juice and control rats [Table 1]. Thirty-eight percent increment of serum ALT level (*P*<0.05) was observed in female SD rats treated for 14 days with star fruit juice after storage for 3 h as compared to control group [Table 2]. However, no significant change of the serum AST, ALP, creatinine and urea was observed in those rats after being administered with 14 days of fresh star fruit juice at different storage times as compared to the control rats [Table 2]. No significant changes in relative organ weight were observed between treatment group and control group [Table 3].

**Table 1:** The effect of star fruit juice on body weight, water intake and food consumption in rats

Groups	Day 0	Day 3	Day 7	Day 14
	**Body weight (g)**			
	
Control	187.97±13.36	193.95±13.88	200.87±16.10	211.62±17.32
T1	185.65±13.86	198.12±18.24	201.39±20.12	210.13±18.41
T2	191.67±11.71	200.65±10.88	199.61±9.47	213.49±10.76
T3	182.96±20.11	191.39±19.61	198.09±19.03	210.20±16.46
	**Water intake (ml/rat/day)**				
	
Control	19.59±4.12	28.75±5.30	32.50±3.54	30.00±7.07
T1	18.34±4.72	29.17±5.89	30.84±1.18	27.09±6.48
T2	23.09±3.42	27.09±0.59	30.42±2.95	28.75±8.84
T3	22.50±3.54	32.09±5.07	28.50±2.12	31.67±9.43
	**Food consumption (g/rat/day)**				
	
Control	12.38±2.83	12.90±2.14	13.58±1.18	13.10±3.10
T1	13.20±2.16	13.17±0.47	13.76±0.08	12.38±0.70
T2	13.39±0.98	14.42±0.19	12.14±5.47	13.48±0.05
T3	14.18±0.52	14.67±1.88	13.95±1.82	14.63±0.04

n=5; Values are expressed in ± S.D; analyzed using Dunnett's test; Control = treated with distilled water only T1 = orally treated with freshly prepared star fruit juice (SFJ); T2 = treated with SFJ after 1 h storage at room temperature; T3 = treated with SFJ after 3 h storage at room temperature

**Table 2:** The effect of star fruit juice on serum AST, ALT and ALP in female SD rats

Groups	Liver Function Tests		
	
	AST (IU/L)	ALT (IU/L)	ALP (IU/L)
Control	145.0±16.02	50.8±9.83	91.4±20.96
T1	130.4±15.34	54.2±13.74	121.0±57.61
T2	151.6±56.93	55.6±6.43	136.4±42.21
T3	132.6±18.45	70.2±9.20*	118.8±39.56
	**Kidney Function Tests**		
	
	**Urea(mg/dL)**		**Creatinine(mg/dL)**

Control	42.6±3.05		0.76±0.09
T1	40.2±4.60		0.76±0.09
T2	36.8±5.76		0.72±0.08
T3	38.0±10.51		0.64±0.13

n=5; values are expressed in mean ± S.D; analyzed using Dunnett's test;

* P<0.05 significantly different as compared to control group; Control = orally treated with distilled water only; T1 = orally treated with freshly prepared star fruit juice (SFJ); T2 = treated with SFJ after 1 h storage at room temperature; T3 = treated with SFJ after 3 h storage at room temperature.

**Table 3:** The effect of star fruit juice on relative organ weight in rats

Groups	Relative Organ Weight (g/100g body weight)				
	
	Heart	Liver	Kidney	Spleen	Lethality
Control	0.37±0.03	2.69±0.25	0.64±0.08	0.19±0.02	0/5
T1	0.38±0.04	2.61±0.26	0.64±0.03	0.21±0.04	0/5
T2	0.39±0.06	2.78±0.23	0.66±0.02	0.22±0.02	0/5
T3	0.36±0.04	2.76±0.26	0.62±0.07	0.21±0.03	0/5

n=5; values are expressed in mean ± S.D; Analyzed using Dunnett's Test; Control = orally treated with distilled water only; T1 = orally treated with freshly prepared star fruit juice (SFJ); T2 = treated with SFJ after 1 h storage at room temperature; T3 = treated with SFJ after 3 h storage at room temperature

The purpose of acute toxicity studies is to find out the total adverse biological effects caused during a finite period of time following the administration of single or several doses repeated over a short interval of time.[9] In toxicity studies, rats and mice are commonly used because they are widely available due to high breeding rate, low cost, small size, ease of handling and the relative abundance of documented toxicological data.[10] The current study clearly provides a general picture or preliminary findings about the toxicity of semi ripened star fruit juice in rats and identifies the targeted organ by star fruit juice by using limited core tests. This study provides important preliminary information about the LD_50_, NOAEL, NOEL and the possible toxic effect of 14 days administration of star fruit juice by conducting an experiment using female SD rats as models.

OECD Test Guidelines 401, conventional acute toxicity test are adopted in the present research. According to OECD Test Guidelines, the volume of single administration dose should not exceed 1 ml/100g (10ml/kg) of body weight except in case of aqueous solution where 2 ml/100g is allowed (OECD guideline 401). According to the star fruit juice preparation, 100 g of semi-ripe star fruit could produce approximately 40 ml of juice. In this study, around 2 ml of star fruit juice was administered per day into rats weighing 200 g for 14 days. Two milliliter of star fruit juice is approximately equivalent to the consumption of 5 g of star fruit daily for rats.

To achieve the objective of the study, several core tests including cage-side observation of death incident, behavioral changes of subjects, body weight change, food intake, water consumption and general blood serum measurements of the common target organs e.g. serum urea and serum creatinine level for renal injury and serum AST, ALT and ALP for hepatic injury were measured. The application of blood serum or plasma enzymes as markers to measure organ damage, cell damage, enzyme induction, activation or inhibition of enzymes become very common in toxicology studies. A variety of blood biochemical measurements could be used to evaluate the severity of tissue damage, possible target organs and measure impaired organ functions. With the combination of these tests, we can evaluate a broad range of information on their physiological and their metabolic functions. Several serum enzymes are used as indicators or markers for hepatocellular injuries such as alanine aminotransferase (ALT) and aspartate aminotransferase (AST).[10] Hepatic “leakage” enzymes are usually cytosolic enzymes. Altered permeability of hepatocellular membrane caused by injury on liver results in the release of soluble cytosolic enzymes into blood.[11] They are generally escaped through basal-lateral side of hepatocytes facing the sinusoids which causing the elevation in blood.[12] Those enzymes are released into blood from the cytosol and subcellular organelles of hepatocytes once liver is injured or damaged. An induction of certain hepatic enzymes is commonly associated with the development of liver damage. Alkaline phosphatase (ALP) and gamma-glutamyltransferase (GGT) are good indicators for hepatobiliary disease. ALP is located mostly within the biliary canaliculi, whereas GGT is mostly in epithelial cells of bile ducts and some periportal hepatocytes. ALP is a group of phosphatase (pH optimum approximately 10) found in almost every tissue in the body. Serum urea level or blood urea nitrogen (BUN) and creatinine test provides information regarding kidney function.[13] Blood nitrogen is part of the urea resulting from catabolism and deamination of amino acid in the liver and it is mainly excreted through urine by kidney. If glomerular filtration rate (GFR) decreases severely which may be caused by renal failure or obstruction of urinary tract, BUN will rise steeply. Creatinine, another indicator of kidney function, is a metabolite of creatine phosphate in skeletal muscle. Its level in blood remains constant normally because the rate of excretion through urine is equivalent to the discharge rate from muscle. If kidney damage or failure is found, the rate of excretion will be lower than the discharge rate from muscle. Thus, the serum levels of urea and creatinine will increase accordingly.[13] However, this observation was not seen in our study although it was previously reported that a single dose administration of star fruit containing 0.2 M oxalate to 8-10 weeks old male SD rats could cause acute renal injury by obstructive effect of calcium oxalate crystals and also by inducing apoptosis of renal epithelial cells.[6] This could be explained by the fact that absorption and distribution of the star fruit juice may vary from rats to rats. Most likely explanation could be that the total amount of oxalate or neurotoxin in star fruit juice that enters to generate serial biochemical reactions in kidney is lower than 0.2 M. On the other hand, the experimental rats may adapt to the repeated dosing by increasing the amount of the hepatic enzyme in their system and changing their physiological systems as well in order to avoid intoxication. Star fruit is a good source of natural antioxidants due to high polyphenolic compound in it, antioxidants could modulate reactive free radicals and reduce its damage to biomolecules.[3,14] Hence, its antioxidant properties may be one of the benefits to counteract the radical damage by oxalate itself.

From the liver function tests, it can be seen that serum ALT level showed 38% increase for rats administered 14 days with star fruit juice after 3 h of storage as compared to the control group. However, serum level of AST and ALP, rats' body weight, food and water intake, relative liver weight did not show any significant changes as compared to the control group. According to the discussion above, increase in ALT actually shows star fruit stored for 3 h mainly targeted the liver. Although it may cause hepatocellular injury, there is no evidence to show that it is toxic to hepatobiliary system as serum ALP level has no significant changes as compared to the control group after administering star fruit juices. In this case, hepatobiliary effect can be ruled out. On the basis of one research reported by Taiwanese researchers, the methanol content in star fruit juice is significantly increased as storage time increases.[7] The same research also reported that star fruit stored for 3 h at 4°C did not produce any significant elevation in terms of methanol content, but it did when stored for 3 h at 30°C. The methanol level investigated after 1 h storage was 5.61 mg/100 ml and after 3 h storage was 14.82 mg/100 ml. The increment of methanol was around 164.17%. Methanol can produce toxic effect to human through ingestion.[6] Methanol is metabolized in liver into formaldehydes, formic acid (toxic), and carbon dioxide by sequential oxidative steps.[14] Accumulation of formic acid and formaldehyde in liver can cause the toxication. Formic acid disrupts mitochondrial electron transport and energy production by inhibiting cytochrome oxidase activity. These can cause depletion of ATP, reduction in essential energy, and finally lead to cell death as essential cell function unable to be maintained. Another mechanism is the formation of cytotoxic reactive oxygen species (ROS) secondary to the blockage of the electron transport chain. ROS causes damage to DNA and other molecules, notably membrane lipids as cells antioxidant defense system of the cells had depleted due to the elevation of oxidative stress.[14] In humans, the signs and symptoms for acute intoxications include headache, vertigo, fatigue, nausea, vomiting, blurred vision, blindness and even death. A dose of pure methanol at 0.1 ml/kg can cause permanent blindness and death, although the lethal dose estimated should be 1-2 ml/kg or 340 μg to 1mg/kg.[7] Elevation of serum ALT alone does not provide sufficient evidence to evaluate the mechanism of star fruit's toxicity, because ALT is also present in skeletal muscle.[15] The damage to skeletal muscle can contribute to its elevation in blood as well. Measurement of creatine kinase is required to rule out skeletal muscle as the origin of ALT. Besides this, further investigation and research as well as histological slides examination can be carried out to check the cause of ALT elevation.

No observable effect level (NOEL) is defined as the highest dose level where no treatment related-findings in any parameter are observed due to treatment. In the present research, NOEL of freshly prepared star fruit juice and that stored for 1 h was established with no significant changes on all tested parameters in the experiment such as body weight change, food and water intake, cage-side observation, serum biochemical markers and others as compared to control group. No observable adverse effect level (NOAEL) is defined as the highest dose level where no adverse treatment related-findings are observed due to treatment. NOAEL of freshly prepared star fruit juice and that stored for 1 h was established with no significant adverse effect observed throughout the experiment, whereas star fruit juice stored for 3 h cannot be considered as NOEL and NOAEL because it elevated the serum ALT level.

From the results of this acute toxicity study, it can be found that LD_50_ values could not be determined because no lethality was found. Therefore, it was believed that the LD_50_ value of star fruit juice is greater than the tested dose in this study. This was supported by the cage-side observations. There was no abnormal behaviour found in all treated rats. Administration of toxicant or chemicals to experimental animals should follow the expected route of administration to the human. In accordance with the modes of administration in humans, star fruit juice is given orally to rats. According to WHO Toxicity Guidelines, 2 weeks to 1 month administration to rats is correlating to a single dose administration or repeated administration for less than one week in human.[16] There are some documented reports to interpret data from one species to another according to their life span, metabolic rate, and total body surface area.[9] In the pharmacokinetic aspect, the metabolism rate in small animals is usually faster than larger animals such as human.[17] For example, the equivalent surface area of the rat's body weight with 0.15 kg to human of 60 kg is about 1/6. Hence, rats are six times more efficient to handle toxic effect as compared to human. If a volume (based on 10 ml/kg) to be fed to man (60 kg), 600 ml of star fruit juice is needed which is also equivalent to 1500 g of star fruit (2 ml juice equivalent to 5 g of star fruit) based on the present juice preparation. Thus, 1500 g divided by 6, is equal to 250 g which is predicted to give the same effect in humans as seen in the present study using rats as model. Therefore, the dose schedule in experimental rats corresponds to an excess intake of star fruit juice in humans. However, it should be borne in mind that the metabolic rate, the rate of enzyme induction and the physiology may be different between humans and rats and many validation works need to be carried out to confirm our suggestions. Controversy is still to be discussed about the conversion factor of equivalent dose from one species to another, but at least this conversion factor provides some correlations between information obtained from animal studies to humans. Results obtained by the preliminary experimental animals study could be used as a consideration in choosing safe doses on humans, to predict the efficacy in therapeutic effects and take precautions if any adverse effects that can affect the physiological system occur.

## CONCLUSION

LD_50_ of the star fruit juice could not be determined but it is suggested to be greater than the tested dose (1ml/100g) in the present study. Fourteen days administration of star fruit extract stored for 1 and 2 h showed no toxic effect either to the liver or kidney functions in young female SD rats. However, star fruit juice stored for 3 h affected liver functions evident by the elevation of serum ALT level in female rats. Further investigation needs to be carried out to confirm the mechanism of toxic effect of star fruit juice in rats.
